# Primary ileal volvulus: a rare twist in an elderly patient—case report

**DOI:** 10.1186/s12893-020-00901-w

**Published:** 2020-10-14

**Authors:** Srikant Agrawal, Ashwini Ranjan Yadav, Bikash Nepal, Pramod Kumar Upadhyay

**Affiliations:** Department of General Surgery, Bir Hospital, National Academy of Medical Sciences, Kathmandu, Nepal

**Keywords:** Ileum, Volvulus, Laparotomy, Case report

## Abstract

**Background:**

Small bowel volvulus is a rare entity and it is even rarer for the ileum to undergo torsion without any known predisposing factors. It presents as acute abdomen with features of intestinal obstruction. As it is a life-threatening condition, it should be kept as a differential for small bowel obstruction despite its rarity. Therefore, we report this case.

**Case report:**

A 60-year-old gentleman presented to our emergency department with a 2-day history of worsening abdominal pain, vomiting, abdominal distension and obstipation. Exploratory laparotomy was done which revealed ileal volvulus with no predisposing factors. Derotation of the segment was done. The postoperative period was uneventful and on follow up after a month, he had a satisfying recovery.

**Conclusion:**

Though primary ileal volvulus is a rare diagnosis, it should be kept in mind in any patient with small bowel obstruction with pain out of proportion and resistant to opioid management. Early diagnosis and urgent surgical intervention is the key to prevent bowel necrosis and associated morbidity and mortality.

## Background

Small bowel volvulus (SBV) refers to the abnormal twisting of a loop of small bowel about the axis of its mesentery [1–4]. This results in partial or complete mechanical intestinal obstruction and eventually to bowel ischemia, necrosis, perforation and peritonitis [5, 6]. SBV is a rare cause of small bowel obstruction accounting 1–4% cases in Western World, but up to 20–35% cases are seen in Asia, Africa and the Middle east [7]. Primary SBV, in which there are no known predisposing factors, is observed mainly in children and young adults, whereas the secondary type is usually found between the age of 40 and 90 years [8, 9]. Central abdominal pain occurring after meal with severity out of proportion to clinical examination and resistant to opioid analgesia should arouse the suspicion of SBV [1, 10]. Diagnosis is difficult at presentation and the investigation of choice is an abdominopelvic computed tomography scan (CT) in which the “whirl sign” can be appreciated [7, 11, 12]. Prompt diagnosis and urgent surgical intervention remain the cornerstone for prevention of bowel necrosis and associated increased morbidity and mortality [7, 12]. We report a case of 60-year-old man in whom early surgical intervention lead to a favorable outcome.

## Case presentation

**Fig. 1:**
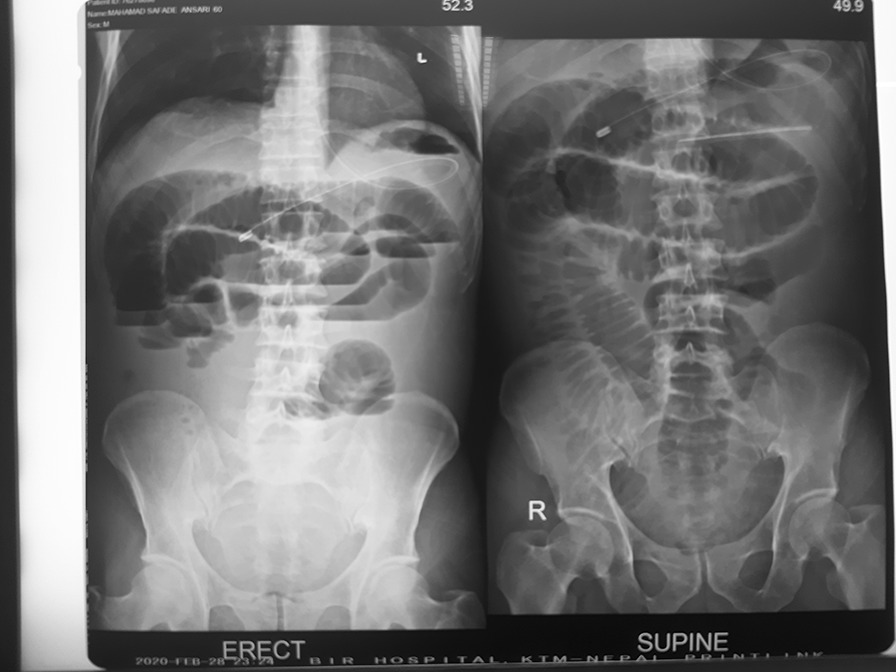
Plain X-ray abdomen showing dilated bowel loops, multiple air-fluid levels and absence of gas in colon

A 60 years old Muslim gentleman with no known co-morbidities presented to our emergency department with a 2-day history of worsening generalized abdominal pain with abdominal distension and absolute constipation associated with few episodes of non-bilious vomiting. There was no history of previous abdominal surgery, fever, per rectal bleeding, unintentional weight loss or similar abdominal pain in past. However, he gave history of ingestion of a large fiber-rich diet after a period of fasting for religious reasons few days back. He had tachycardia of 110 beats per minute with the remaining vital signs within normal limit and on examination of the abdomen; generalized abdominal distension with tenderness and rebound tenderness was noted. Bowel sounds were sluggish and per-rectal examination was normal. Blood investigations revealed normal hemogram with hyponatremia. Plain X-ray of abdomen showed multiple air-fluid levels, dilated proximal small bowel loops and absence of gas in colon (Fig. 1). An abdominal ultrasound revealed multiple dilated bowel loops with interbowel loop collection without any definite radiological sign.

**Fig. 2:**
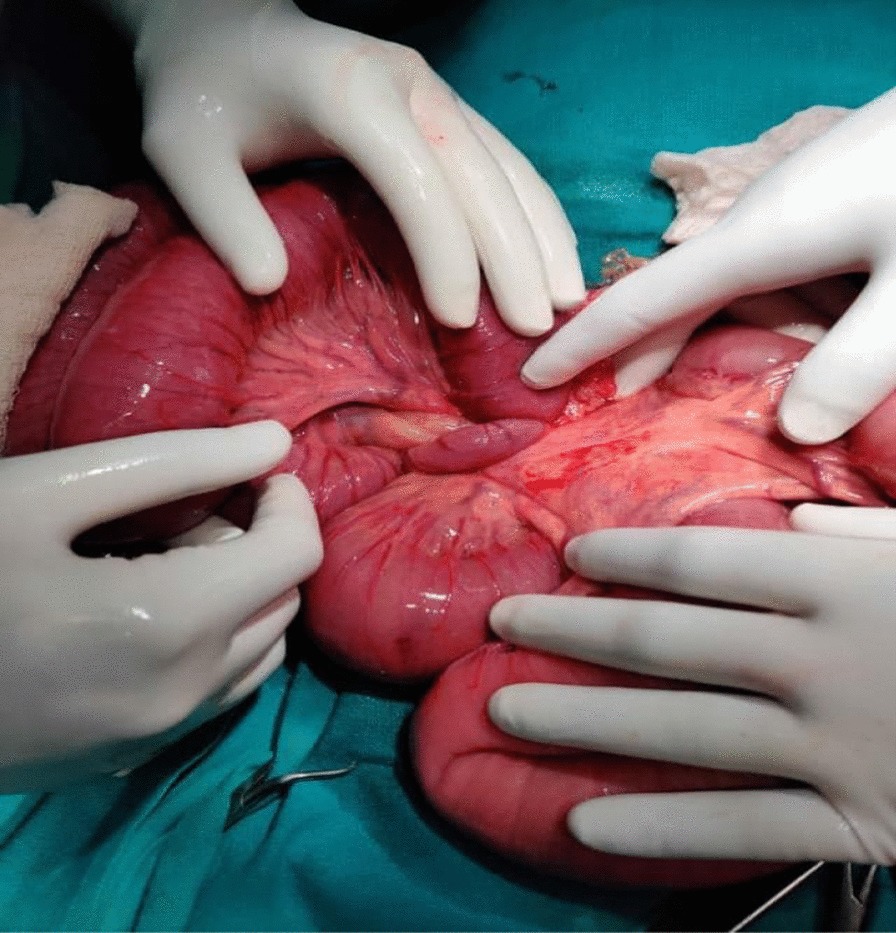
Intraoperative photograph showing volvulus of ileum

A clinical diagnosis of complete bowel obstruction was made, the patient was resuscitated and exploratory laparotomy was performed with a midline incision. Minimal reactive ascitic fluid was present. It was noted that there was 720° anticlockwise rotation of a 20 cm segment of the distal ileum around its mesentery forming a volvulus about 35 cm proximal to the ileocaecal junction (Fig. 2). Bowel loops proximal to the site of obstruction were dilated and the distal loops were collapsed. Rest of the bowel was healthy. Untwisting of the volvulus was done by rotating the segment in clockwise direction, the dilated bowel loops were decompressed and the abdomen was closed. The post-operative period was uneventful and he was discharged on the fourth postoperative day. At a follow up in Outpatient Department after 30 days, our patient was doing fine with no further abdominal symptoms.

## Discussion and conclusion

Volvulus is a cause of dynamic intestinal obstruction resulting from torsion of a loop of bowel around the axis of its own mesentery [1, 2, 4]. While colon is the most common site of volvulus, it is seldom seen in small bowel [1]. Primary ileal volvulus is rare and only few cases have been reported [2]. The annual incidence of SBV is 1.7–5.7 cases per 100,000 population in North America and western Europe, but rates as high as 24–60 cases per 100,000 population have been reported in Africa, Asia and the Middle East, thought to be related to dietary habits such as ingestion of a large volume of fiber after prolonged period of fasting in Muslim communities [3, 7, 11, 13].

On the basis of etiology, SBV can be primary or secondary. Volvulus is considered to be primary in the absence of predisposing factors and is most commonly seen in children and young adults, whereas secondary SBV occurs in the setting of an underlying lesion upon which the mesentery can rotate. Various theories have been postulated to explain the occurrence of primary SBV such as long mobile mesentery with a shorter mesenteric base, strong abdominal muscles, increased peristaltic tone and ingestion of a large volume of fiber-rich diet after prolonged period of fasting. Volvulus may occur secondary to adhesions, fibrous band, Meckel’s diverticulum, congenital malrotation of gut, tumors, mesenteric lymph nodes, parasitic infestations, internal hernias, lipomas, pregnancy, endometriosis and hematomas [1, 2, 4, 9, 10]. During laparotomy, no secondary cause was identified in our patient even after thorough exploration of the abdomen. Parasitic infestations were not seen in his stool test on follow up. In the absence of an identifiable pathology, ingestion of large fiber-rich meal after prolonged fasting may have predisposed our patient to volvulus.

The most common presentation is that of an acute abdomen with central abdominal pain out of proportion to the physical findings and resistant to narcotic analgesia along with features of small bowel obstruction [1, 2, 4, 10]. The duration of vascular insult is directly related to the severity of abdominal pain rather than the degree of intestinal obstruction [13].

Plain abdominal radiographs are nonspecific and fail to demonstrate the cause of small bowel obstruction [1, 4]. Contrast-enhanced CT Scan of abdomen pelvis is the investigation of choice with findings such as the “whirl sign”, “spoke wheel sign”, “beak sign” and “barber pole signs” suggests the diagnosis of SBV and features like presence of air in the wall of bowel, free peritoneal fluid and portal vein gas point towards intestinal ischemia [7, 14, 15]. The sensitivity of CT is reported up to 80%, however none of the findings is pathognomic [7, 11, 14]. Our patient presented with worsening abdominal pain, generalized tenderness and rebound tenderness. Plain abdominal radiographs revealed features of complete bowel obstruction. Taking this emergency setting in account with the diagnosis of complete bowel obstruction in a virgin abdomen, CT abdomen was not done and the patient was shifted to the operation theatre for emergency laparotomy.

Emergency surgery is the treatment to be undertaken with the aim of untwisting the volvulus and re-establishing the blood flow [6]. If the bowel is necrotic, resection is needed but the management of primary SBV cases with viable bowel is controversial, the options being either resection or simple derotation, with or without fixation of the involved bowel [4, 6]. Resection in viable bowel cases results in longer hospital stays with increased morbidity while derotation alone can result in recurrence rates up to 30% [6, 11]. Surgical fixation of small bowel is technically challenging and its long term results are not documented [6]. For secondary SBV, the underlying cause should be addressed during laparotomy [1, 6]. In our case, simple derotation was done, the loaded bowel loops were decompressed and on thorough exploration of the abdomen, no secondary cause was identified. Fixation of the small bowel was not done due to paucity of literature about its effectiveness and long term effects. On follow up after 30 days, the patient had no abdominal symptoms.

Depending on the time delay before surgical intervention, the mortality rate of SBV ranges from 9 to 35% but with gangrenous bowel, rates as high as 20–100% have been reported [3, 13].

In spite of being a rare entity, primary ileal volvulus should be kept in differential for small bowel obstruction. Early diagnosis and urgent surgical intervention remain the cornerstone for prevention of bowel necrosis and associated increased morbidity and mortality.

## Data Availability

Secondary data were collected from the patient’s hospital records.

## Abbreviations

SBV: Small bowel volvulus
CT: Computed tomography

## References

[1] Islam S, Hosein D, Dan D, Naraynsingh V (2016). Volvulus of ileum: a rare cause of small bowel obstruction. BMJ Case Rep.

[2] Faizan S, Jain AKC, Thimmappa D (2020). Primary ileal volvulus: a rare cause of small intestinal obstruction. IntSurg J.

[3] Roggo A, Ottinger LW (1992). Acute small bowel volvulus in adults: a sporadic form of strangulating intestinal obstruction. Ann Surg.

[4] Klein J, Baxstrom K, Donnelly S, Feasel P, Koles P (2015). A fatal twist: volvulus of the small intestine in a 46-year-old woman. Case Rep Med.

[5] Bernstein S, Russ P (1998). Midgut volvulus: a rare cause of acute abdomen in an adult patient. Am J Roentgenol.

[6] Tam A, Phong J, Yong C (2019). Primary small bowel volvulus: surgical treatment dilemma. ANZ J Surg.

[7] Lepage-Saucier M, Tang A, Billiard JS, Murphy-Lavallée J, Lepanto L (2010). Small and large bowel volvulus: clues to early recognition and complications. Eur J Radiol.

[8] Huang JC, Shin JS, Huang YT, Chao CJ, Ho SC, Wu MJ (2005). Small bowel volvulus among adults. J GastroenterolHepatol.

[9] Grasso E, Sciolli L (2011). Spontaneus small bowel volvulus in an adult: case report and review of the literature. Ann ItalChir.

[10] Patial T, Chaddha S, Rathore N, Thakur V (2017). Small bowel volvulus: a case report. Cureus.

[11] Ruiz-Tovar J, Morales V, Sanjuanbenito A, Lobo EM (2009). Volvulus of the small bowel in adults. Am Surg.

[12] Katis PG, Dias S (2004). Volvulus: a rare twist on small-bowel obstruction. CMAJ.

[13] Iwuagwu ODG (1999). Small bowel volvulus: a review. J R CollSurgEdinb.

[14] Li XB, Guan WX, Gao Y (2010). Multislice computed tomography angiography findings of chronic small bowel volvulus with jejunal diverticulosis. Jpn J Radiol.

[15] Rudloff U (2005). The spoke wheel sign: bowel. Radiology.

